# Improving community health worker use of malaria rapid diagnostic tests in Zambia: package instructions, job aid and job aid-plus-training

**DOI:** 10.1186/1475-2875-7-160

**Published:** 2008-08-22

**Authors:** Steven A Harvey, Larissa Jennings, Masela Chinyama, Fred Masaninga, Kurt Mulholland, David R Bell

**Affiliations:** 1 University Research Co., LLC, 7200 Wisconsin Ave., Suite 600, Bethesda, MD, 20814, USA; 2 Malaria Consortium, Post Net Box 748, P/Bag E 891, Lusaka, Zambia; 3 World Health Organization – WHO Zambia Office, UN Annex, Plot No. 4609, Andrew Mwenya/Belt Rds, P.O. Box 32346, Rhodes Park, Lusaka, Zambia; 4 World Health Organization – Regional Office for the Western Pacific, P.O. Box 2932, Manila, Philippines

## Abstract

**Background:**

Introduction of artemisinin combination therapy (ACT) has boosted interest in parasite-based malaria diagnosis, leading to increased use of rapid diagnostic tests (RDTs), particularly in rural settings where microscopy is limited. With donor support, national malaria control programmes are now procuring large quantities of RDTs. The scarcity of health facilities and trained personnel in many sub-Saharan African countries means that limiting RDT use to such facilities would exclude a significant proportion of febrile cases. RDT use by volunteer community health workers (CHWs) is one alternative, but most sub-Saharan African countries prohibit CHWs from handling blood, and little is known about CHW ability to use RDTs safely and effectively. This Zambia-based study was designed to determine: (i) whether Zambian CHWs could prepare and interpret RDTs accurately and safely using manufacturer's instructions alone; (ii) whether simple, mostly pictorial instructions (a "job aid") could raise performance to adequate levels; and (iii) whether a brief training programme would produce further improvement.

**Methods:**

The job aid and training programme were based on formative research with 32 CHWs in Luangwa District. The study team then recruited three groups of CHWs in Chongwe and Chibombo districts. All had experience treating malaria based on clinical diagnosis, but only six had prior RDT experience. Trained observers used structured observation checklists to score each participant's preparation of three RDTs. Each also read 10 photographs showing different test results. The first group (n = 32) was guided only by manufacturer's instructions. The second (n = 21) used only the job aid. The last (n = 26) used the job aid after receiving a three-hour training.

**Results:**

Mean scores, adjusted for education, age, gender and experience, were 57% of 16 RDT steps correctly completed for group 1, 80% for group 2, and 92% for group 3. Mean percentage of test results interpreted correctly were 54% (group 1), 80% (group 2), and 93% (group 3). All differences were statistically significant (p < 0.05).

**Conclusion:**

Manufacturer's instructions like those provided with the RDTs used in this study are insufficient to ensure safe and accurate use by CHWs. However, well-designed instructions plus training can ensure high performance. More study is underway to determine how well this performance holds up over time.

## Background

Widespread introduction of artemisinin combination therapy (ACT) has generated renewed interest in parasite-based diagnosis of malaria. This, in turn, has led to an increase in the use of malaria rapid diagnostic tests (RDTs), particularly in rural settings where functional microscopy is limited [1,2]. National malaria control programmes are now procuring large quantities of RDTs with support from The Global Fund to Fight AIDS, Tuberculosis and Malaria (GFATM), the U.S. Presidential Malaria Initiative (PMI), and other donors. There are currently more than 80 commercially available RDTs, most targeting *Plasmodium falciparum *[3]. Unlike traditional microscopy, RDTs detect malaria parasite antigen in finger-stick blood samples; they do not require microscopes or other laboratory equipment. Yet despite their relative simplicity, RDT accuracy is highly user-dependent. Poor test preparation and interpretation can result in incorrect diagnoses. This, in turn, can lead to unnecessary antimalarial use, failure to address the real cause of fever in patients who do not have malaria, and withholding of treatment from patients who do [4-11].

Parasite-based diagnosis is essential for good management of febrile illness in malaria-endemic areas. However, in many such areas, more than half of febrile patients seek treatment at the community level without ever visiting a health facility [12]. Thus, limiting RDTs to health facilities would greatly reduce the number of febrile cases diagnosed using a parasite-based method. In some areas of Latin America and Asia, volunteer community health workers (CHWs) have offered community-based testing for many years: in earlier times by preparing blood smears for microscopic diagnosis; more recently using RDTs [13]. CHW use of RDTs was tested on a small scale in Tanzania as early as 1993 [14], but concerns about accidental transmission of blood-borne diseases including HIV have made many African health systems reluctant to permit blood handling by CHWs.

Wider RDT use by CHWs could facilitate parasite-based malaria diagnosis in settings with limited health personnel and facilities. This paper reports on a study designed to determine whether Zambian CHWs – supported by a job aid and brief training programme – could prepare and interpret malaria RDTs accurately and safely. Job aids are verbal or pictorial instructions that – when combined with training or supervision – enhance a health worker's ability to correctly perform specific tasks [15,16]. Recent studies have shown that job aids can improve accuracy of RDT preparation among health personnel with minimal training [17,18].

## Methods

### Study area and population

The study was conducted in July 2006 in Lusaka Province, Zambia. The study area is endemic for *P. falciparum *malaria, and laboratory diagnostic services are available to only a third of febrile patients [19]. All CHWs were observed at the health centre nearest their home village.

### Sample selection

**Figure 1:**
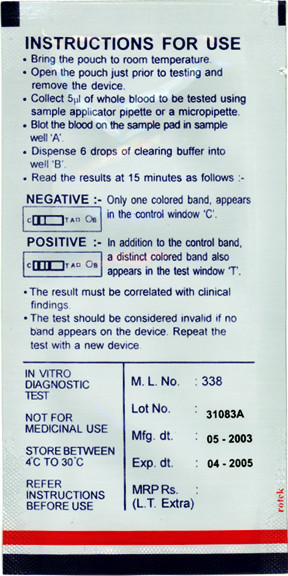
**Manufacturer's instructions for the Paracheck Pf^® ^rapid diagnostic test**. Actual size: 6.6 × 13 cm

**Figure 2:**
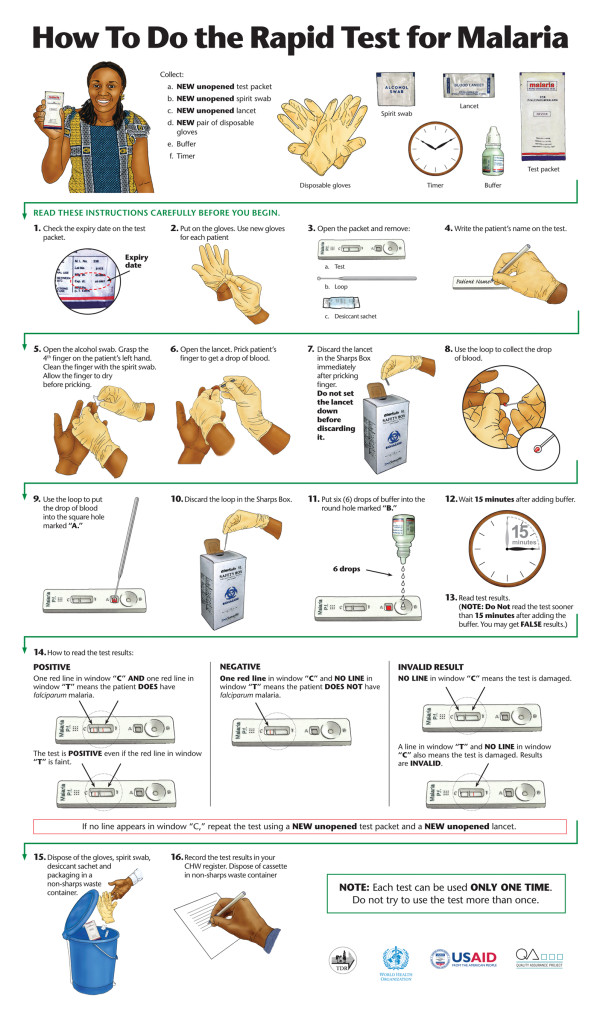
**Final English version of the job aid for malaria RDT preparation**. Actual size: 594 × 841 cm (A1 sheet)

To test the effect of job aid and job aid-plus-training on CHW performance, the study team recruited three independent groups of CHWs. In the first group, CHWs prepared the RDT using only the manufacturer's instructions (Figure 1). These instructions were provided in both English and Nyanja, the local language. In group 2 CHWs used only the job aid (Figure 2), also provided in English and Nyanja. In group 3, CHWs prepared the RDT using the job aid after receiving three hours of training. All CHWs used the Paracheck Pf^® ^rapid test device for *P. falciparum *malaria (Orchid Biomedical Systems, India). CHWs for each group were recruited based on availability and ability to reach testing sites with patient volume sufficient for each CHW to see three subjects within a few hours. The target sample was 23 CHWs per group (total 69), sufficient to detect a mean difference of 20% between groups at 90% power. At the time of the study, all participating CHWs lived in Chongwe or Chibombo District.

### Formative research

The job aid and training programme were based on formative research with 32 CHWs carried out in January 2006 in Luangwa District. The formative research began with nine focus groups. In each group, a researcher from the Zambia National Malaria Control Centre (NMCC) demonstrated RDT preparation and interpretation. A local facilitator then asked participants to suggest how best to explain the test to other CHWs, comment on what steps CHWs might find difficult, and recommend how best to overcome these difficulties. Based on these findings, the study team designed a draft job aid. A second round of focus groups was conducted to get feedback on the draft, which was then modified to incorporate CHW recommendations. This modified job aid was then used to conduct the present study.

### Training

**Figure 3:**
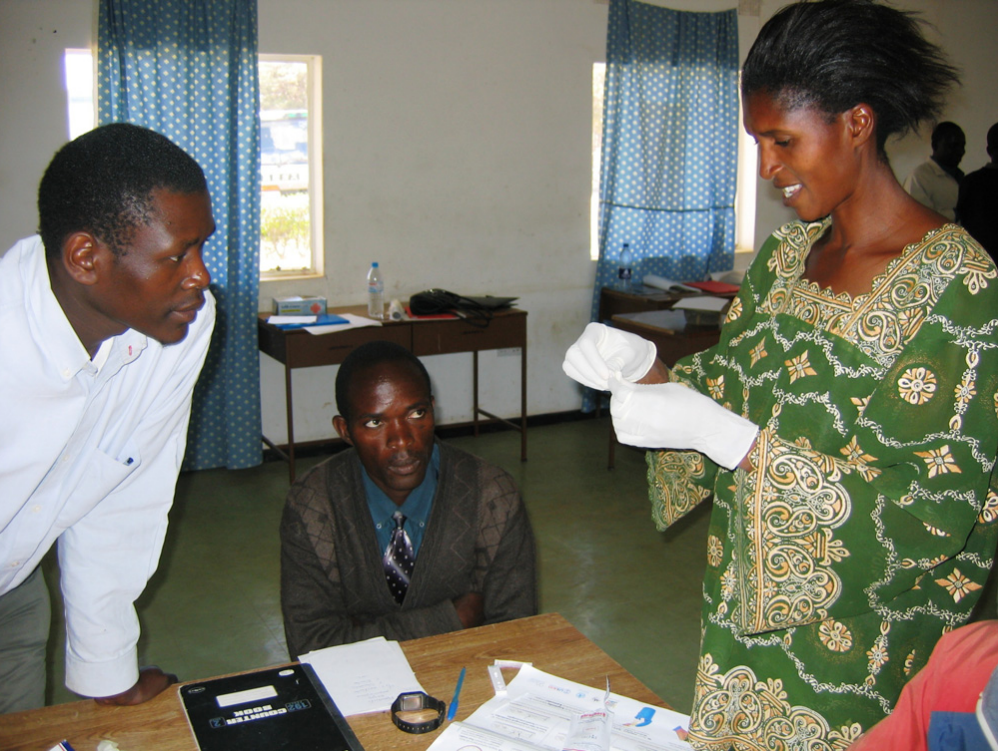
**CHW training in RDT preparation**. As an observer (left) and another CHW (center) watch, a Zambian CHW prepares to open a blood lancet during training on RDT use.

**Figure 4:**
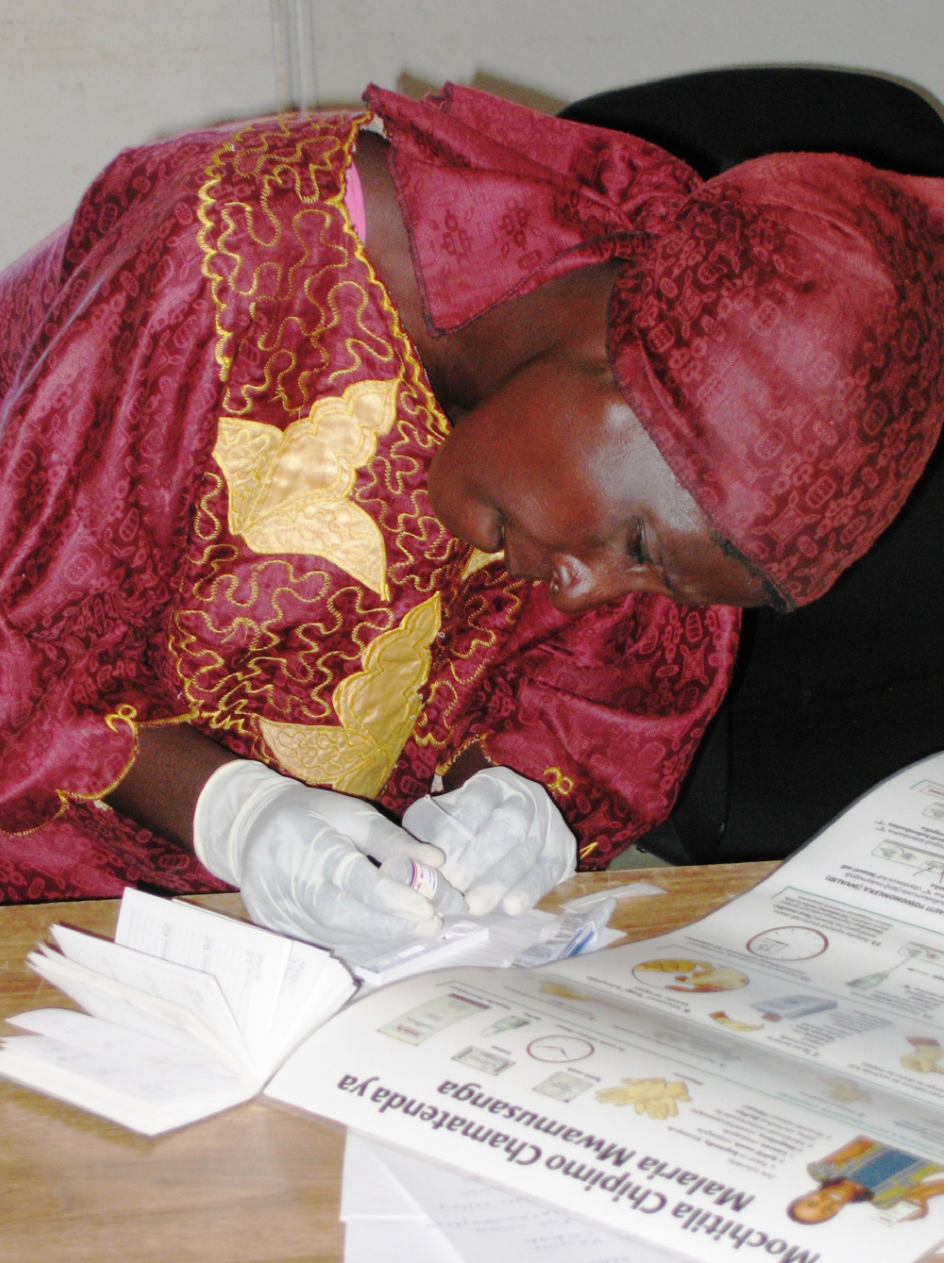
**CHW training in RDT preparation**. A Zambian CHW adds buffer to a malaria rapid diagnostic test during training on RDT use.

CHWs in the study's training arm participated in a three-hour course in RDT preparation. First, a trainer demonstrated step-by-step how to carry out the test, from opening the test packet to reading the results. Next the trainer presented a module focused on appropriate finger-pricking technique. Participants then practiced the test on one another and received coaching from the trainer and several experienced assistants (Figures 3 and 4). Before conducting tests on actual patients, all participants had to demonstrate competency in practice sessions. Finally, the trainer or an assistant quizzed each CHW using photographs to ensure all participants could distinguish between strong positive, faint positive, negative, and invalid results.

A study objective was to test whether CHWs could achieve satisfactory competence with a minimal investment of time and resources. The training required no equipment. Necessary supplies included RDTs, gloves, alcohol swabs, lancets, sharps and non-sharps bins, and a copy of the job aid for each participant. The study also covered transportation costs and one night's lodging for CHWs who lived too far away to return home the day of the training. Altogether, these materials totalled about US $66.00 per CHW trained. Including salaries, per diem, and transportation costs for the trainer, observers, and MOH personnel raises the total per CHW cost to slightly less than US $175.00.

A generic version of the job aid and the training materials used in this study may be downloaded from the WHO's malaria RDT website [20], which also provides information on adapting these materials for different products and contexts.

### Measurement

This study measured two outcomes: (1) ability to conduct test procedures safely and correctly and (2) ability to interpret the results correctly.

To assess CHW ability to conduct test procedures, local observers used a 16-item checklist based on discrete steps necessary to prepare and interpret the diagnostic test. Each CHW was observed preparing and interpreting RDTs on three different febrile patients. For each test, observers noted whether the CHW performed each step correctly, incorrectly, or not at all. The findings reported here cover each CHW's third test. Since most participants had never used an RDT prior to the study, the authors hypothesized that the third test would represent a more accurate measure of each CHW's ability to perform than would the first two.

To analyse specific aspects of test procedures, the authors grouped the 16 steps into three sub-categories: (1) preparation and documentation, (2) RDT use and (3) safe handling and disposal. The first included steps related to preparing the patient, assembling materials and recording results. The second included steps essential for test performance: checking the test expiry date, collecting and transferring blood, adding buffer and waiting a sufficient time (15 minutes) before reading test results. The last included use of clean gloves and a sterile lancet as well as proper disposal of sharps and bio-hazardous materials. Observers noted any specific errors or difficulties for each step. The mean percentage of steps performed correctly was calculated for the entire test and for each sub-category.

To assess CHW ability to interpret RDT results, each participant read a photograph of 10 tests with a combination of positive, negative, and invalid outcomes. The percentage of tests correctly interpreted by each CHW was then calculated. Data on participant age, gender, education, home district, years working as a CHW, prior experience treating malaria and prior experience using RDTs were also obtained. All data were entered into Microsoft Access.

### Data analysis

Data were analysed using Stata version 9.2 (Statacorp, College Station, TX). Paired t-tests were used to assess differences in overall scores between each CHW's first, second and third RDT. Bivariate and multivariate linear regression was utilized to examine between-group differences (manufacturer's instructions vs. job aid-only; and job aid-only vs. job aid-plus-training) in total and sub-category scores.

A core assumption of linear regression is that data follow a normal distribution [21]. To compensate for the non-normal distribution of CHW test scores in this study, the authors employed bootstrapping for re-sampling with replacement, using 1,000 replications. Bootstrapping has been shown to provide high accuracy for a variety of non-normal distributions [22,23]. Two-sample tests of proportions were used to compare between-group performance on an item-by-item basis. In all analyses, *p *values ≤ 0.05 were considered statistically significant.

### Ethics approval

This study received ethics approval from WHO/TDR and the Tropical Disease Research Centre Ethics Committee – Ndola, Zambia. Because Zambia had not approved routine RDT use by CHWs at the time of the study, all febrile patients received presumptive treatment, regardless of RDT status.

## Results

### Participant characteristics

A total of 81 CHWs were recruited for the study: 32 in the manufacturer's instructions group, 23 in the job aid-only group, and 26 in the job aid-plus-training group (Table 1). Two CHWs in the second category were excluded from analysis due to inability or unwillingness to participate, leaving 21 job aid-only participants. Most CHWs (90%) had prior malaria treatment experience, but few (8%) had prior RDT experience. There were no significant differences in CHW characteristics by group with the exception of education level. Over a third (35%) of CHWs in the job aid-plus-training group had completed secondary education, compared to 6% and 19% in the manufacturer's instructions and job aid-only groups respectively (p < 0.05).

**Table 1:** Socio-demographic characteristics of community health workers (n = 79)

CHW characteristics [n, (%)]	Manufacturer's instructions (n = 32)	Job aid only (n = 21)	Job aid plus training (n = 26)	Total (N = 79)
Gender (women)	6 (18.8%)	8 (38.1%)	6 (23.1%)	20 (25.3%)
Mean age (years)	41.6	41.3	43.6	42.2
Mean years as CHW	6.0	5.1	5.6	5.6
Completed secondary education*	2 (6.3%)	4 (19.1%)	9 (34.6%)	15 (19.0%)
Chongwe district	18 (56.3%)	11 (52.4%)	11 (42.3%)	40 (50.6%)
Prior malaria treatment experience	27 (84.4%)	20 (95.2%)	24 (92.3%)	71 (89.9%)
Prior RDT experience	3 (9.4%)	1 (4.8%)	2 (7.7%)	6 (7.6%)

* Significant at *p *< 0.05.

### Accuracy of test procedure

On average, CHWs using the manufacturer's instructions performed 57% of test steps correctly. Those using the job aid alone improved significantly to 80%. Job aid-plus-training CHWs scored highest at 90% correct. In multivariate analyses, none of the CHW characteristics significantly affected overall performance. Table 2 presents both adjusted and unadjusted total scores and sub-category scores.

**Table 2:** Mean scores by category and total for RDT performance by CHWs using manufacturer's instructions, job aid-only, and job aid-plus-training (N = 79)

	**Manufacturer's instructions vs. Job aid-only**			**Job aid-only vs. Job aid plus training**		
		
	% of steps performed correctly		Difference β (95% CI)	% of steps performed correctly		Difference β (95% CI)
	Manufacturer's instructions (n = 32)	Job aid- only (n = 21)		Job aid- only (n = 21)	Job aid plus training (n = 26)	
*Unadjusted scores^a^*						

Mean correct skill steps (total)	57	80	+23 (13, 33)*	80	90	+10 (3, 17)^a^*
Mean correct skill steps (by category)						
Preparation and documentation	49	69	+20 (7, 34)*	69	83	+14 (1, 27)*
RDT use	61	89	+28 (16, 39)*	89	92	+3 (-4, 10)
Safe handling and disposal	58	80	+22 (7, 36)*	80	95	+15 (5, 25)*
Mean correct RDT readings (total)	54	82	+28 (20, 36)*	82	93	+11 (3, 18)*

*Adjusted scoress^a,b^*						

Mean correct skill steps (total)	57	80	+23 (12, 34)*	80	92	+12 (3, 20)*
Mean correct skill steps (by category)						
Preparation and documentation	52	72	+20 (6, 35) *	72	89	+17 (4, 30) *
RDT use	60	88	+28 (16, 40) *	88	92	+4 (-5, 13)
Safe handling and disposal	56	77	+21 (6, 37) *	77	92	+15 (4, 26) *
Mean correct RDT readings (total)	54	80	+26 (17, 34)*	80	93	+13 (4, 22)*

[a] Based on observations of the 3^rd ^febrile patient; [b] Adjusted for CHW educational status, age, gender, years working as a CHW, prior experience using an RDT, and prior experience in malaria treatment. * Significant at *p *< 0.05.

By sub-category, job aid-only CHWs improved in all areas compared to those using manufacturer's instructions. In sub-category 1 (preparation and documentation), unadjusted mean scores improved from 49% to 69% of steps performed correctly. In sub-category 2 (RDT use), unadjusted mean scores rose from 61% to 89%. In sub-category 3 (safe handling and disposal), unadjusted mean scores rose from 58% to 80%.

Supplementing the job aid with training further improved CHW performance. In preparation and documentation, the unadjusted mean score rose from 69% for the job aid-only group to 83% for the trained group. In the safe handling and disposal sub-category, scores were 80% in the job aid-only group and 95% in the trained group. There was no difference between job aid-only and trained CHWs for steps in the "RDT use" category. As with the total scores, all differences remained significant after controlling for CHW characteristics.

At the item level, there was considerable variation in the percentage of steps completed correctly (Table 3). The largest improvements were in checking the expiry date, cleaning the patient's finger, collecting blood, disposing of sharps and non-sharps materials, and documenting test results. Performance of tasks such as removing test packet contents and using a sterile lancet was generally satisfactory among the manufacturer's instructions group and showed no significant gains in the job aid-only and job aid-plus-training groups.

**Table 3:** Item analysis of test steps by category: preparation and documentation, RDT use, and safe handling and disposal for CHWs using manufacturer's instructions, job aid-only, and job aid-plus-training (N = 79)

**Test steps (organized by category)**	**% participants who performed step** ** correctly**			**Difference (%) (95% CI)**	
		
	Manufacturer's instructions (n = 32)	Job aid only (n = 21)	Job aid- plus-training (n = 26)	Manufacturer's instructions vs. Job aid-only	Job aid-only vs. Job aid-plus- training
*Preparation and documentation*					

1. Assembles packet, buffer, swab, lancet	48	86	85	+37 (14, 60)*	-1 (-22, 19)
2. Removes contents of test packet	91	90	85	-1 (-17, 16)	-5 (-24, 14)
3. Writes patient's name on cassette	16	76	77	+61 (38, 83)*	+1 (-24, 25)
4. Selects 4^th ^finger on left hand; cleans it with alcohol swab, and allows it to air dry	26	57	96	+31 (5, 58)*	+39 (17, 61)*
5. Records results in CHW register	62	38	73	-24 (-51, 3)	+35 (8, 62)*
Sub-total for category:	49	69	83	+20 (7, 34)*	+14 (1, 27)*

*RDT Use*					

1. Checks expiry date on test package	21	67	96	+46 (21, 71)*	+29 (8, 51)*
2. Collects film of blood with enclosed loop making sure to fill the loop completely	63	90	73	+28 (7, 49)*	-17 (-39, 4)
3. Using the loop, blots blood on the pad in sample well A	72	95	100	+23 (5, 41)*	+5 (-4, 14)
4. Dispenses six drops of clearing buffer into well B	81	100	96	+19 (5, 33)*	-4 (-11, 4)
5. Waits 15 minutes before reading results	70	90	92	+20 (0, 41)	+2 (-14, 18)
Sub-total for category:	61	89	92	+28 (16, 39)*	+3 (-4, 10)

*Safe handling and disposal*					

1. Puts on new pair of gloves	66	86	96	+20 (-2, 42)	+10 (-6, 27)
2. Using a sterile lancet, punctures finger	88	86	96	-2 (-21, 17)	+10 (-7, 27)
3. Discards lancet in sharps bin immediately after pricking finger. Does not set it down first.	41	62	96	+21 (-6, 48)	+34 (12, 56)*
4. Discards the loop in the sharps box	55	90	100	+36 (14, 57)*	+10 (-3, 22)
5. Disposes of gloves, wrappers, alcohol swab, loop, desiccant and cassette in non-sharps container	45	76	85	+31 (6, 56)*	+8 (-14, 31)
Sub-total for category:	58	80	95	+22 (7, 36)*	+15 (5, 25)*

*Interpretation of results*					

1. Reads test results correctly	72	86	96	+13 (-9, 35)	+10 (-6, 27)

Total (all 16 steps)	57	80	90	+23 (13, 33)*	+10 (3, 17)*

* Significant at *p *< 0.05.

### Errors in RDT use

Table 4 lists commonly observed test preparation errors and difficulties. CHWs using manufacturer's instructions often had difficulty identifying test materials, understanding package instructions, and collecting or transferring blood. They also omitted key tasks such as cleaning the patient's finger or adding buffer. On occasion, CHWs used alcohol (indicated for cleaning the patient's finger) as buffer or an alcohol swab to collect and transfer blood. These errors were not observed among CHWs in the job aid-only or the job aid-plus-training groups.

**Table 4:** Observer reported errors and difficulties by CHW group (not listed by frequency)

Test category and steps	Manufacturer's instructions	Job aid-only	Job aid plus training
***Preparation and documentation***			
1. Assembles materials 2. Removes contents from test packet 3. Records patient's name on cassette 4. Selects 4th finger of left hand, cleans it with alcohol swab, allows it to dry 5. Records test results in CHW register	- Did not record test results - Recorded results prior to completion of test - Difficulty identifying test packet contents and their use - Difficulty understanding instructions - Prepared incorrect finger	- Lacked CHW register - Prepared incorrect finger	- Lacked CHW register - Did not assemble contents prior to test

***RDT Use***			
1. Checks expiration date to make sure test is still valid 2. Collects film of blood with enclosed loop making sure to fill the loop 3. Using the loop, blots blood on the pad in sample well A 4. Dispenses six drops of clearing buffer into well B 5. Waits 15 minutes before reading results	- Difficulty drawing adequate quantity of blood - Difficulty using loop for collection of blood - Transferred blood to incorrect well - Omitted use of buffer - Omitted use of loop - Collected blood with lancet - Used alcohol as buffer - Used alcohol swab to collect and transfer blood - Left swab or other materials in test well - Did not monitor time - Waited > 15 minutes prior to reading results - Did not check test expiry date	- Difficulty drawing adequate quantity of blood - Difficulty using loop for collection of blood - Transferred blood in incorrect well - Dispensed buffer prior to placing blood - Did not monitor time - Did not check test expiry date	- Difficulty drawing adequate quantity of blood - Difficulty using loop for collection of blood - Waited > 15 minutes - Did not check test expiry date

***Safe handling and disposal***			
1. Puts on new gloves 2. Uses sterile lancet to prick finger 3. Discards lancet in sharps bin immediately after pricking 4. Discards loop in the sharps bin immediately after transferring blood to test cassette 5. Disposes of gloves, wrappers, alcohol swab, loop, desiccant, and cassette in non-sharps bin	- Omitted cleaning finger prior to pricking - Punctured finger multiple times - Lancet set down on table and re-used on same patient - Incorrect disposal of items in sharps vs. non-sharps container - Near use of same pair of gloves on new patient - Near use of same RDT on new patient	- Punctured finger multiple times - Lancet set down on table and re-used on same patient - Incorrect disposal of items in sharps vs. non-sharps container - Near use of same pair of gloves on new patient	- Punctured finger multiple times - Incorrect disposal of items in sharps vs. non-sharps container

Some participants in all groups experienced difficulty drawing or collecting an adequate volume of blood from the first puncture. This led some CHWs to puncture patients' fingers multiple times. These errors were less frequent among job aid-only and job aid-plus-training participants. At times, CHWs in all groups were unsure about which items should be disposed of in sharps versus non-sharps containers. Errors such as transferring blood to the incorrect test well or placing a used lancet on the table before discarding it were observed in the manufacturer's instructions and job aid-only groups, but not in the trained group. Failure to check the expiry date was observed in all three groups, though less frequently among job aid-only and job aid-plus-training participants.

### Accuracy of test interpretation

Accuracy of test interpretation improved significantly in the job aid-only and job aid-plus-training groups in both unadjusted and adjusted models (Table 2). Manufacturer's instructions CHWs read a mean 54% of test results correctly compared to 82% in the job aid-only group and 93% in the job aid-plus-training group. The most common mistake was to read a faint positive or invalid result as negative. Occurrences of this error declined significantly from a mean of 2.3 in the manufacturer's instructions group to 1.7 in the job aid-only group and again to 0.3 in the job aid-plus-training group (p < 0.05). In the manufacturer's instructions group, no CHW correctly read all 10 test results compared to 7 (33%) in the job aid-only and 16 (62%) in the trained group (p < 0.05).

### Influence of practice on performance

Performance also improved with each successive RDT prepared by a CHW (data not reported). Pair-wise total scores from the second practice test were significantly higher than those from the first (mean difference: 7%, 95% confidence interval [CI]: 3–11%); and scores from the third test (the one observed and reported on here) were significantly higher than those from the second (mean difference: 4%, 95% CI: 2–6%). Differences between consecutive tests were larger in the manufacturer's instructions and job aid-only groups than in the job aid-plus-training group.

## Discussion

Results of this study indicate that CHWs can prepare and interpret malaria RDTs correctly and safely when supported by clear instructions and appropriate training. Conversely, malaria control programmes cannot expect adequate performance if they rely solely on manufacturer's package instructions like those provided with the RDTs used in this study. In fact, sole reliance on manufacturer's instructions as currently designed will likely result in high levels of misdiagnosis and mismanagement, putting CHWs, patients and the community at risk. CHWs using the job aid had significantly higher scores than those relying on manufacturer's instructions both overall and in all sub-categories (preparation, RDT use, safety and interpretation of results). The training further improved scores overall and in the sub-categories of preparation, safety and interpretation. Safety errors observed in the manufacturer's instructions or job aid-only groups were less frequent or absent in the training arm. Similarly, interpretation of faint positive and invalid results improved considerably when job aids were supplemented with training.

Training group scores were not significantly higher in the "RDT use" sub-category. As shown in Table 3, ≥ 90% of job aid-only participants correctly performed four of the five steps in this sub-category. However, only 67% of job aid-only participants checked the test expiry date compared to 96% in the job aid-plus-training group. This, plus the higher scores in the other sub-categories, suggests that training is necessary to ensure satisfactory performance.

Practicing RDT preparation was also associated with improved performance. The increase in scores for each successive test highlights the value of practice for skill acquisition. However, practice alone did not produce satisfactory performance: the competency gains associated with repetition were much smaller than those associated with either the job aid alone or the job aid plus training. Only CHWs in the latter group demonstrated consistently satisfactory results.

The study identified three potential concerns regarding CHW use of RDTs. The first relates to performing the finger prick and collecting and transferring blood to the test. While this study did not directly measure the blood volume transferred, qualitative assessment suggests that CHWs in all groups sometimes obtained too little or too much blood. Most participants had never taken a finger prick blood sample before the study and had some difficulty with their initial attempts.

The most commonly observed problem was inadequate puncturing technique. Rather than using a stabbing motion, some CHWs would set the point of the lancet on the patient's fingertip and try to push it in. Others would stab too lightly. Both cases often resulted in too small a volume of blood. A related problem was difficulty in expressing blood from the fingertip. When an initial puncture failed to produce a sufficient volume, some CHWs would squeeze both sides of the fingertip towards the centre or squeeze from too close to the puncture, thus constricting rather than augmenting blood flow.

**Figure 5:**
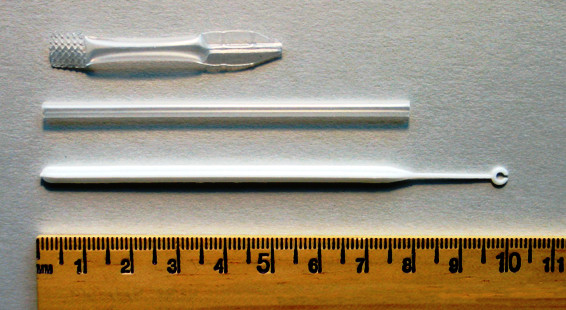
**Blood collection devices packaged with various RDTs**. Plastic blood collection loop enclosed with the Paracheck Pf^® ^rapid diagnostic test used in this study (bottom) and plastic straw and pipette packaged with other RDTs. All three have caused difficulties for some users.

The blood collection device included with the test used in this study was a plastic loop about 2 mm in diameter attached to a plastic handle about 10 cm long (Figure 5). The instructions directed users to collect a thin film of blood across the opening of the loop, which was designed to hold 5 μl. This proved difficult for some participants. Holding the fingertip facing up and attempting to collect the blood from above tended to yield a thin coating of blood around the edges of the loop, but not a film across the opening – an insufficient volume. Though not described in the manufacturer's instructions, a more successful technique was to hold the finger tip and blood drop facing downward and collect the blood from underneath. Once professional health workers discovered this technique through trial and error, it was incorporated into the job aid and training for CHWs. Other blood collection devices (Figure 5) have caused similar problems.

High variability in the amount of blood transferred by CHWs is cause for concern: inadequate volume can reduce sensitivity while excess volume may cause background staining and obscure faint results [24]. CHW training in RDT use should include practice with blood collection devices to ensure that CHWs master the appropriate technique. Manufacturers should work to improve the design of blood collection devices to reduce the risk of error.

The second concern relates to reading test results too soon, perhaps because the package instructions give insufficient emphasis to the importance of waiting. Previous study findings support this conclusion [18]. As shown in Figure 1, the package says, "Read the results at 15 minutes as follows:" then shows monochrome line drawings illustrating negative and positive outcomes. In this study, focus group results indicated that the combination of words used in steps 12 and 13 of the job aid, plus the image of a minute hand moving through a 15-minute interval on a clock face (Figure 2), communicate the concept more effectively. RDT manufacturers should consider building a timing device into the test itself or providing a low-cost timer along with each box of tests to reduce possible misunderstandings. Anecdotally, more and more CHWs seem to have mobile phones, and the diffusion of mobile technology into even the remotest areas may soon make other timing devices unnecessary.

The last concern is incorrect interpretation of test results. CHWs in the manufacturer's instructions and job aid-only groups frequently read faint positive or invalid tests as negative. In the first case, the strength of the test line can vary significantly depending on level of parasitaemia, blood viscosity, volume of blood and other factors [25]. In this study, training to recognize faint results proved beneficial. However, ambient lighting conditions or poor eyesight may compromise ability to distinguish faint positive from negative results even after instruction. A few studies have shown poor visual acuity and limited ability to afford glasses at a population level in sub-Saharan Africa [26,27]. The authors were unable to find studies focused specifically on Zambian or other CHWs, and this study did not test participants' visual acuity, but it seems reasonable to assume that as a group, their vision and access to corrective eyewear are no better than average. If this assumption is correct, distinguishing a faint positive result in dim light (e.g., at night by kerosene lamp or candlelight) could be quite challenging.

Misreading invalid test results is a somewhat different issue: while both the manufacturer's instructions and the job aid mention that a test line in the absence of a control line or no line at all means the test is invalid, it is easy to misconstrue this instruction to mean "line = positive, no line = negative." CHW interpretation of faint positive and invalid results improved considerably in the job aid-plus-training group. If RDTs are to be used for widespread community-based diagnosis, low-cost aids to improve visibility (such as lighting or eyewear) as well as training to recognize faint results are likely to enhance CHW performance and confidence in reading test results. Training to recognize invalid test results is also critical.

While some amount of training seems critical to ensuring adequate performance, lengthy training programmes can strain scarce health system resources both human and financial. Multi-day trainings take professional health workers away from patients and volunteer CHWs away from income-producing activities and family responsibilities. Health systems bear the cost of materials, equipment, lodging, transportation, food, and often additional per diems. In this study, training was kept short to test whether CHWs could achieve satisfactory competence with a minimal investment. At least in the short term, the results seem quite satisfactory.

Training need not be costly or extensive, but basic principles of educational psychology suggest that demonstration, practice and feedback are crucial to mastering the motor skills involved in performing a good finger prick or collecting blood and transferring it to a rapid test [28]. Given study findings, the authors recommend more emphasis on blood collection and transfer. Providing effective demonstration, practice and feedback requires a trainer-trainee ratio small enough that a trainer or experienced assistant can observe and coach each participant. More research is needed to determine the ideal ratio, but pending such research, the authors recommend no more than about 12 participants for a team including one trainer and 2 or 3 assistants.

## Limitations

As always, the limitations of this research must be considered when interpreting study findings. Data came from two districts near Lusaka, so it is possible that participating CHWs were better educated and had access to more information, supplies and support than would a CHW from a more distant region. Furthermore, participants were not randomly selected, and most were observed in health centres rather than in the community where they typically work. Though all off-site participants were reimbursed travel expenses, those from the most distant villages were under-represented. Assessment of CHW performance using manufacturer's instructions is based solely on the package instructions provided with Paracheck Pf^© ^when this study took place. Performance using other instructions may vary. Ministries of health and others considering community-based malaria case management should note that the training programme designed for this study focused strictly on RDT use. Training in management of both parasite-positive and parasite-negative patients would need to be provided separately. Further research is now underway to determine how well performance observed immediately after training will hold up over time.

## Conclusion

Use of malaria rapid diagnostic tests by community health workers is potentially an effective alternative for malaria case management in areas with limited functional microscopy and limited health care personnel or facilities. Findings from this study show that a well-designed job aid and brief training can ensure high CHW performance. Addressing design issues related to time-keeping, visibility and blood sampling may further enhance RDT preparation and interpretation by CHWs and the resultant quality of case management.

## List of abbreviations

ACT: Artemisinin combination therapy; AusAID: Australian Agency for International Development; CHW: Community health worker; CI: Confidence interval; GFATM: Global Fund to Fight AIDS, Tuberculosis and Malaria; HIV: Human immunodeficiency virus; NMCC: Zambia National Malaria Control Centre; PMI: United States Presidential Malaria Initiative; RDT: Rapid diagnostic test; TDR: WHO Special Programme for Research and Training in Tropical Diseases; USAID: United States Agency for International Development; WHO: World Health Organization.

## Competing interests

The authors declare that they have no competing interests.

## Authors' contributions

SH was the principal investigator, designed the study, supervised and participated in data collection and analysis, and participated in drafting and revising the manuscript. LJ analysed the data and wrote and revised substantial portions of the manuscript. MC coordinated and supervised field work for both phases of the study, participated in data collection, was principally responsible for designing and carrying out the training programme, and contributed to the manuscript. She worked for the Zambian National Malaria Control Centre at the time of the study activities described here. She now works for the Malaria Consortium in Lusaka. FM coordinated local and regional WHO participation in and support for the study. He participated in study design and data collection and contributed to the manuscript. KM participated in both phases of data collection, designed the graphics and artwork for the job aid and training manual, and contributed to the manuscript. DB conceived the study and participated in design and data collection. He coordinated regional and global WHO support for and participation in study activities and provided substantial contributions to the manuscript. All authors read and approved the final draft.
